# Identification of Two Novel Fluorinases From *Amycolatopsis* sp. CA-128772 and *Methanosaeta* sp. PtaU1.Bin055 and a Mutant With Improved Catalytic Efficiency With Native Substrate

**DOI:** 10.3389/fbioe.2022.881326

**Published:** 2022-06-13

**Authors:** Xinming Feng, Yujin Cao, Wei Liu, Mo Xian

**Affiliations:** ^1^ CAS Key Laboratory of Biobased Materials, Qingdao Institute of Bioenergy and Bioprocess Technology, Chinese Academy of Sciences, Qingdao, China; ^2^ University of Chinese Academy of Sciences, Beijing, China

**Keywords:** catalytic efficiency, site-directed mutagenesis, organic fluoride, biocatalysis, fluorinase

## Abstract

Fluoride plays an important role in the fields of materials and medicine. Compared with chemical synthesis, fluorinases are natural catalysts with more application potential, which provide a green and effective way to obtain organofluorine. However, the application of fluorinases is limited by certain factors, such as the limited number of enzymes and their low activity. In this work, two new fluorinases from *Amycolatopsis* sp. CA-128772 and *Methanosaeta* sp. PtaU1.Bin055 were identified by gene mining and named Fam and Fme, respectively. The activities of these two enzymes were reported for the first time, and Fme showed good thermal stability, which was different from the reported fluorinases. In addition, the activity toward natural substrate of Fam was improved by site-directed mutagenesis, the catalytic efficiency (*k*_*cat*_ */K*_*m*_) of the best mutant containing two amino acid substitutions (T72A and S164G) toward the substrate S-adenosyl-L-methionine was improved by 2.2-fold compared to the wild-type. Structural modeling analysis revealed that the main reason for the increased enzyme activity might be the formation of a new substrate channel. Experimental evidence suggests that the substrate channel may indeed play a key role in regulating the function of the fluorinases.

## 1 Introduction

Fluorine is getting increasing attention in the chemical industry because of its unique properties (Zhu et al., 2018). The incorporation of fluorine in the molecule will lead to the formation of many beneficial properties, such as reactivity, selectivity, biological activity, and physical properties (Walker and Chang, 2014; Yuan et al., 2017). This is especially true in the pharmaceutical industry and the material industry (Cros et al., 2022). Fluorine substitution has become an effective means of designing bioactive compounds (Meanwell, 2018). To date, approximately 25% of drugs on the market contain at least one C-F bond (Purser et al., 2008). Incompatible with its importance is the scarcity of natural products of fluoride, fluorine chemistry is almost a synthetic field (Furuya et al., 2011; Carvalho and Oliveira, 2017). Due to the extremely strong electronegativity of fluorine, conventional fluorination reactions often require highly toxic fluorinated agents or harsh reaction conditions (Liang et al., 2013). By contrast, using fluorine-containing building blocks to prepare complex organofluorine is a more efficient synthetic strategy.

Fluorinase, which catalyzes S-adenosyl-_L_-methionine (SAM) and potassium fluoride to form 5′-fluoro-5′-deoxyadenosine (5′-FDA) and _L_-methionine, provides a green option for the efficient preparation of fluorine-containing compounds. The first discovered fluorinase was FlA from *Streptomyces cattleya* (O'Hagan et al., 2002). Due to the ability to work in water under mild conditions, fluorinase has been developed for the preparation of [^18^F]FDA from SAM and [^18^F]F^−^, which can be used in positron emission tomography (PET) for pathologic diagnosis (da Silva et al., 2018). The metabolic pathway of *S. cattleya* involved with fluorinase has been identified, and a variety of intermediate products have been shown to be the effective pharmaceutical precursors (O’Hagan and Deng, 2015). For example, fluoroacetic acid has been used as a building block to synthesize fluoropolyketone compounds, while another product, 4-fluorothreonine, is considered to be a valuable compound in fields of the biosciences for producing fluorinated peptides (Odar et al., 2015; Thuronyi and Chang, 2015). In general, fluorinase is the most critical enzyme in the fluorination pathway because it is responsible for the organization of fluoride ions, but only five new fluorinases have been discovered and characterized (Deng et al., 2014; Wang et al., 2014; Ma et al., 2016; Sooklal et al., 2020).

At present, studies on the biological conversion of fluoride mainly include the exploration of new fluorinases and the modification of metabolic pathways that can utilize fluorinated building blocks as a precursor (Wu et al., 2020). Recently, an interesting study reported by Calero et al. (2020) showed that organofluorine biosynthesis can be implemented in the platform bacterium *Pseudomonas putida* with inorganic F^−^ as the only fluorine source. Among these fluorinases that have been characterized, most of them show a low turnover number (Deng et al., 2014). Obtaining the fluorinase with higher catalytic efficiency is critical to understand the catalytic mechanism of enzymes and also beneficial for their applications. Sun et al. (2016) identified two FlA1 mutants with increased activity for 5′-chloro-5′-deoxyadenosine. However, fluorinase with enhanced catalytic activity toward SAM has not been reported. Herein, we report two novel fluorinase sequences and modify their activities.

## 2 Materials and Methods

### 2.1 Materials

Isopropyl β-D-thiogalactopyranoside (IPTG), 5′-fluorodeoxyadenosine (5′-FDA), S-adenosyl-L-methionine (SAM), and potassium fluoride (KF) were obtained from commercial corporations (Aladdin, Sigma-Aldrich, Macklin, etc.). The Mut Express II Fast Mutagenesis Kit V2 was obtained from Vazyme Biotech Co. All the primers used were synthesized by Tsingke Biotechnology Co.

### 2.2 Sequence Homology Searches for Novel Fluorinases

To identify novel fluorinases, the FlA from *S. cattleya* with NCBI access no. Q70GK9.1 was selected as the reference sequence. The 21 amino acid motif between residues 90R and 111E of FlA, which was recognized as the characteristic information of fluorinases, was set as the query sequence (Sooklal et al., 2020).

### 2.3 Recombinant Plasmid Construction and Expression in *E. coli*

pETDuet-1 plasmid and *E. coli* strain BL21 (DE3) or Rosetta (DE3) were used for gene expression. After optimization for the *E. coli* expression system, the gene sequences encoding candidate proteins were synthesized by Tsingke Biological Technology and connected with pETDuet-1 plasmid between the BamHI and HindIII restriction sites. The *E. coli* cells containing expression vector were grown in LB broth (pH7.0, Oxoid), with 100 μg/ml ampicillin at 37°C until the OD_600_ reached about 0.8. Then the target protein was overexpressed at 30°C for 6 h upon induction with 0.4 mM ITPG.

### 2.4 Enzyme Purification

The induced cells were harvested at 5,000 rpm at 4°C and washed twice with Tris-HCL buffer (pH 7.8, 50 mM). Then, the cells were disrupted in a constant system cell breaker (Constant Systems) using a pressure setting of 30 kpsi. The supernatant (crude enzyme) was collected by centrifugation (Hitachi) at 12,000 rpm for 20 min at 4°C and purified by Ni-NTA His Bind Resin (Sangon Biotech). Tris-HCl (20 mM, pH 8.0) containing 30 mM imidazole was set as the wash buffer, while the elution buffer contained 250 mM imidazole. Then the eluate was concentrated using the tubular ultrafiltration modules (Millipore Merck, 10 kDa) at 4,500 rpm and repeatedly washed with Tris-HCl buffer to remove imidazole. The protein concentration was determined by the Bradford Protein Assay Kit.

### 2.5 Enzyme Assays

Typically, the assay was carried out in Tris-HCl buffer (pH 7.8, 50 mM), containing SAM (0.4 g/L), KF (100 mM) with crude (total protein 400–500 μg/ml), or purified enzyme (50–75 μg/ml) at 50°C. The assay was stopped by a water bath at 98°C for 2 min. All the samples were detectable by HPLC with UV detection. An enzyme unit was defined as the amount of enzyme producing 1 μmol of 5′-FDA per minute.

### 2.6 Effect of Temperature

To explore the impact of temperature on the candidate fluorinase, the assay was carried out at various temperatures from 4 to 60°C. Relative activity of 100% was defined as the highest rate of 5′-FDA formation, and other enzyme activities were calculated as relative activities.

To explore the thermostability of the candidate fluorinase, purified enzymes were incubated at different temperatures for 30 min, and then the substrates were added to the assay solution for residual activity determination.

### 2.7 Kinetic Assays of the New Enzymes

To determine the kinetic parameters of the enzymes, the assays were carried out in Tris-HCl buffer (pH 7.8, 50 mM), containing 200 mM KF with SAM (60–1,200 µM) at optimized temperature for 10 min and stopped by a water bath at 98°C for 2 min. The initial rate of the reaction was calculated based on the amount of product produced.

### 2.8 Homology Modeling and Site-Directed Mutagenesis

The structure of the candidate proteins was constructed on the Swiss-model system (https://swissmodel.expasy.org/) by homology modeling with 2V7V as the template. The quality of the model was evaluated by a Ramachandran plot.

In order to screen the key sites of the enzyme, single-point mutations were constructed by replacing hot amino acids with alanine using Mut Express II Fast Mutagenesis Kit V2. Then saturation mutation at the key sites was used to obtain single-point mutants with increased activity. The double mutant was obtained by combining single-point mutants. All the primers used in the site-directed mutagenesis process were designed using the CE tool (vazyme.com) with a Tm of about 67°C (Supplementary Table S1).

### 2.9 Structural Modeling and the Molecular Dynamics Simulation Method

Based on the structure of Fam, the T72A/S164A mutant was constructed using the amino acid mutation method, and the structure of the substrate SAM and F^−^ ion was obtained from the crystal structure (PDB ID: 2V7T). Molecular dynamics (MD) simulation was carried out using the Gromacs 2018.4 program (Van der Spoel et al., 2005), which was carried out under constant temperature and pressure and periodic boundary conditions. The Amber99SB all-atom force field and the TIP3P water model were adopted (Jorgensen et al., 1983). In the MD simulation process, all hydrogen bonds involved were constrained by the LINCS algorithm (Hess et al., 1997), and the integration step was set to 2 fs. Electrostatic interaction was calculated by the particle-mesh Ewald (PME) method (Darden et al., 1993). The cutoff value for non-bonding interactions was set to 10 Å, and was updated every 10 steps. The V-rescale temperature coupling method was used to control the simulated temperature to 300 K, and the Parrinello–Rahman method was used to control the pressure to 1 bar (Berendsen et al., 1984; Martoňák et al., 2003). First, the steepest descent method was used to minimize the energy of the two systems to eliminate the close contact between the atoms. Then, a 100 ps NVT equilibrium simulation was performed at 300 K. Finally, the two systems were subjected to 50 ns of MD simulation with the conformation saved every 10 ps.

## 3 Results and Discussion

### 3.1 Selection of the Fluorinase Gene

**FIGURE 1:**
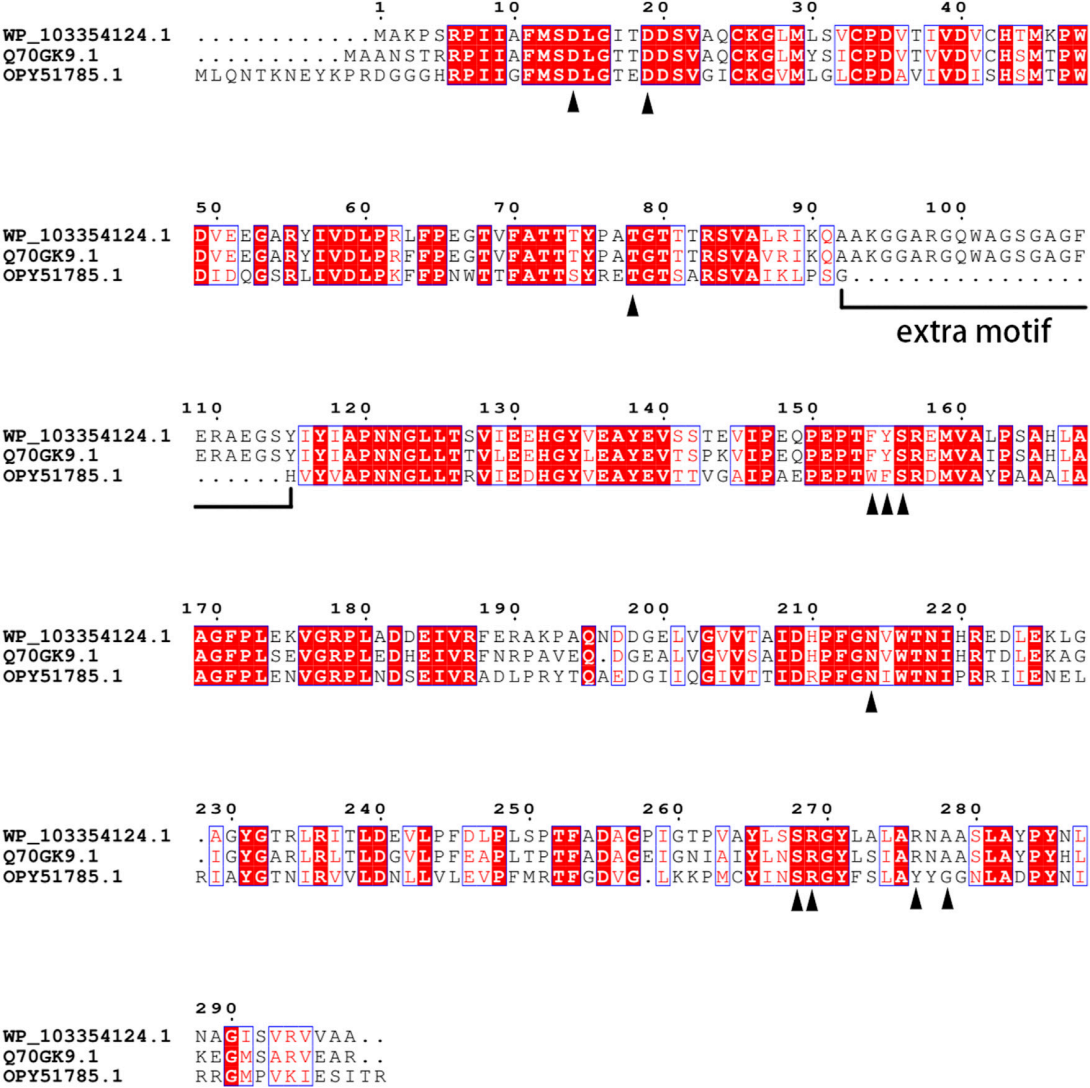
Alignment of multiple amino acid sequences of FlA, Fme, and Fam. WP_103354124.1: Fam. Q70GK9.1: FlA. OPY51785.1: Fme. The amino acids in direct contact with the substrate were identified by the black triangle.

To identify novel candidate fluorinases, a homology-based database screening was conducted with FlA as the template. According to previous studies, all the fluorinases have an extra motif at a specific position, and the amino acid sequences at this motif have high similarity (Deng et al., 2014; Sooklal et al., 2020). Dong et al. (2004) reported that this motif may affect the quaternary structure of fluorinase . Taking this into account, a protein sequence (WP_103354124.1) from *Amycolatopsis* sp. CA-128772 showed 82% identity of the FLA was selected as a candidate protein because it contained an identical extra motif. The active sites of FlA have been speculated, and the result shows that 11 amino acid residues (16D, 21D, 80T, 156F, 157Y, 158S, 215N, 269S, 270R, 277R, and 279A) may be involved in the hydrogen bonding and electrostatic interaction between the enzyme and the substrate (Senn et al., 2005). Sequence comparison revealed that these 11 amino acid residues were conserved in the sequence of Fam (Figure 1). Deng et al. (2006) reported that the FlA is also a chlorinase, which led us to wonder whether chlorinase might also be a fluorinase . Then, the protein sequence labeled “chlorinase” (OPY51785.1) from *Methanosaeta* sp. PtaU1. Bin055 was selected because of the high sequence similarity with FlA (50.34% identity), although it does not contain the extra motif, let alone is not completely consistent on some key residues.

The gene of Fam was found in contig0000085 of the *Amycolatopsis* sp. CA-128772 whole genome shotgun sequence (NCBI accession: PPHG01000085.1). Analysis of the DNA sequence and manual BLAST searching at the GenBank database showed that seven genes around the fluorinase gene were highly conserved comparing with gene clusters in *S. cattleya*, the coded functions include DNA regulation (FlG5, FlL5, FlJ5, and FlF5), 5′-FDA phosphorylase (FlB5), S-adenosyl-L-homocysteine hydrolase (FlI5), and transporter protein (FlH5) (Supplementary Table S2). The gene Fme was located at contig000143 of the *Methanosaeta* sp. PtaU1.Bin055 whole genome shotgun sequence (NCBI accession: MVRM01000143.1). Gene sequence analysis showed that FlK6 may encode fluoroacetyl-CoA thioesterase, which was also highly conserved in *S. cattleya* (Supplementary Table S3).

### 3.2 Production and Activity Verification of Fluorinases

The candidate proteins were expressed using BL21 (DE3). The cells were lysed and centrifuged, and the pure enzyme was then extracted from the soluble components. The theoretical sizes of Fme and Fam are 33.9 and 34.1 kDa, respectively. Both proteins were well expressed in *E. coli* to produce an intense band on SDS-PAGE gels after programmed production and purification (Supplementary Figure S1).

**FIGURE 2:**
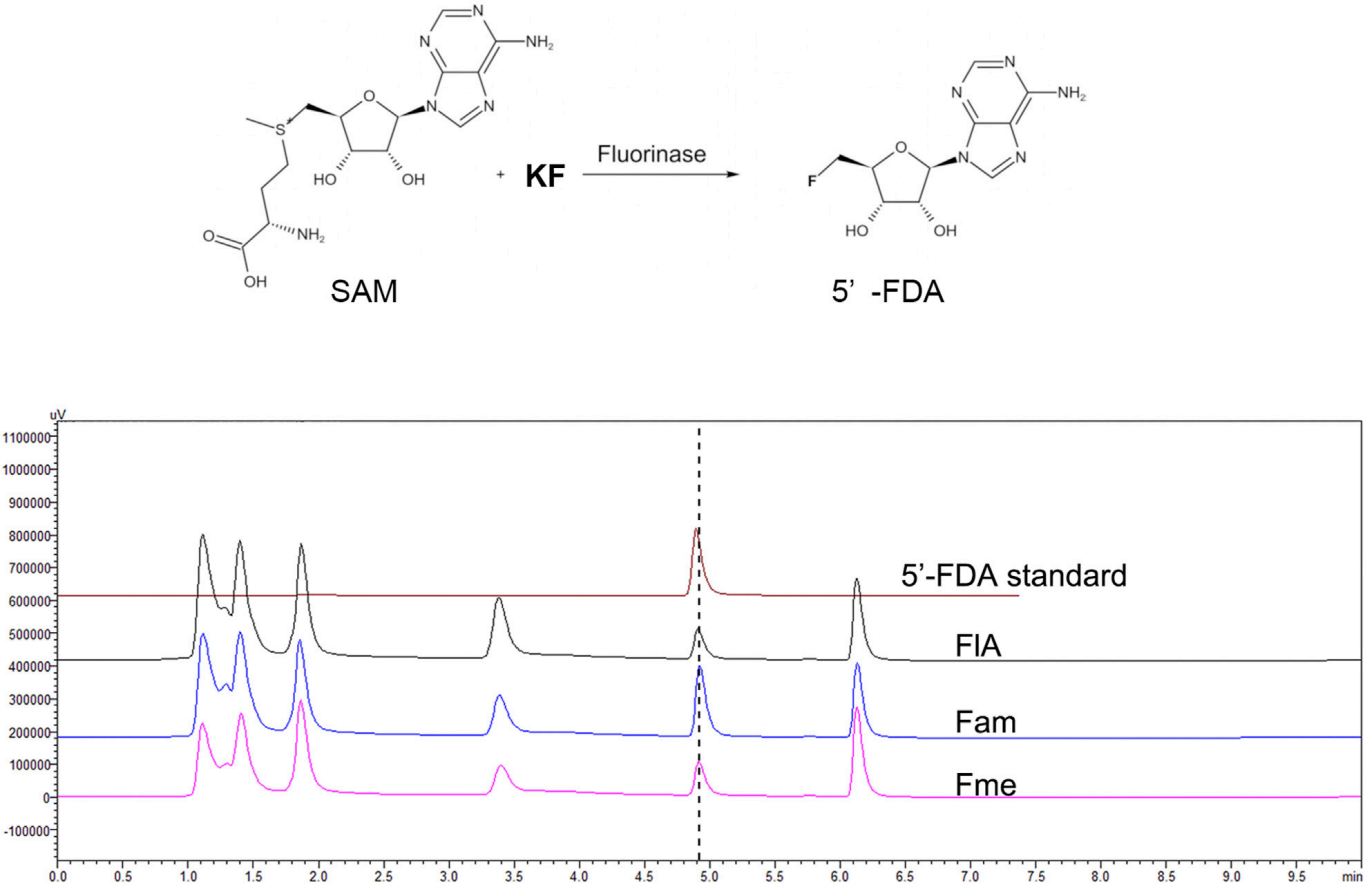
HPLC analysis of the products in different samples. The assays were carried out with Tris-HCl (50 mM, pH 7.8) containing 200 mM KF, 1 mM SAM, and 500 μg/ml crude enzyme at 50°C for 2 h.

Fluorinases catalyze the reaction between SAM and F^−^ to form 5′-FDA, which can be detected by HPLC. To verify the function of the candidate proteins, the assays were carried out with the crude enzyme (Figure 2). The HPLC analysis of catalytic samples of Fme, Fam, and FlA (fluorinase from *S. cattleya*) confirmed the existence of the 5′-FDA, HPLC-MS was also used to confirm the presence of 5′-FDA in the samples (Supplementary Figure S2). The results indicated that both candidate proteins had the function of fluorinase. It is worth mentioning that a higher concentration of 5′-FDA was detected in the sample of Fam, which may indicate better enzyme activity.

Different from the reported enzymes, Fme also has the function of fluorinase, although no extra motif is present in the protein sequence. A recent study proves that the hexameric form of fluorinase is not necessary for catalytic activity, which explains the rationality of Fme activity (Kittilä et al., 2022). According to previous reports, fluorinase can catalyze the chlorination reaction, while chlorinase cannot produce organic fluoride by using F ions. Therefore, we believe that Fam named labeled “chlorinase” in NCBI should be fluorinase. In order to explore the effect of the extra motif on the function of Fam, the 91–112 region of Fam was deleted to obtain Fam0, and the activity of the modified protein was verified by HPLC. The results showed that Fam0 was expressed in *E. coli*, but no activity was detected (Supplementary Figure S3). The loss of enzymatic activity might be due to the impaired enzyme structure.

### 3.3 Effect of Temperature

**FIGURE 3:**
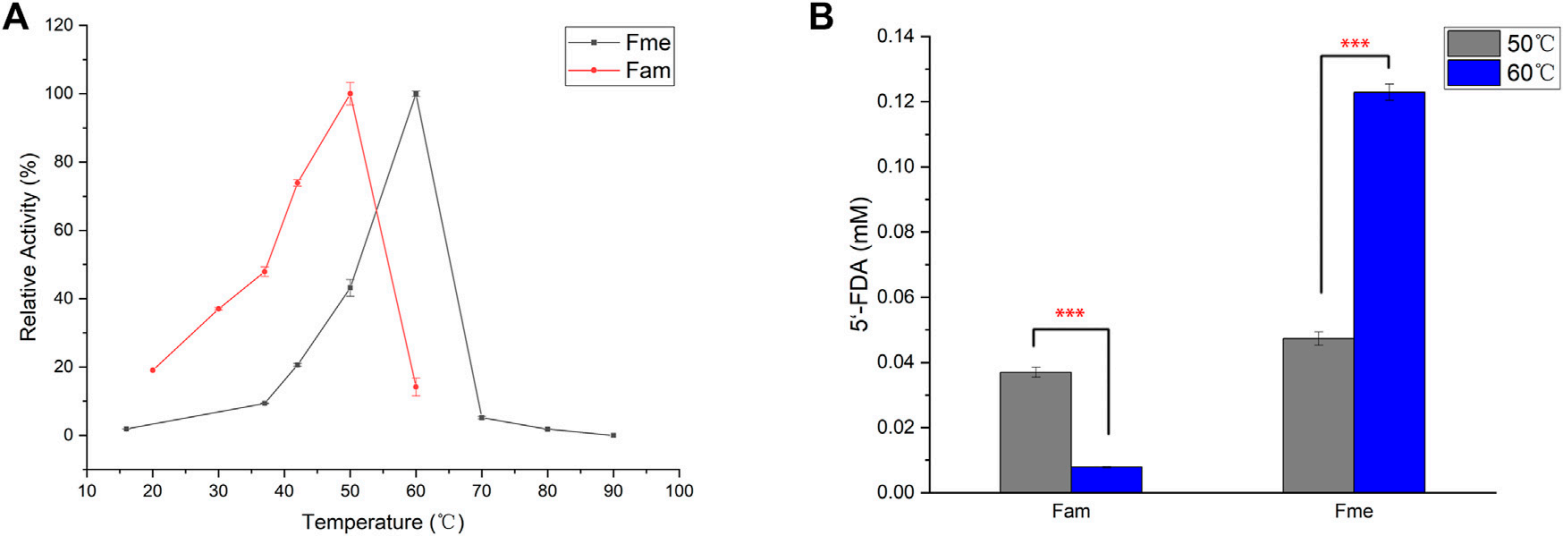
Influence of temperature on fluorinase. **(A)** Effect of temperature on fluorinase activity. Assays were carried out in Tris-HCl buffer (50 mM, pH 7.8), containing KF (100 mM), SAM (400 µM), and enzyme (60 μg/ml) for 1 h **(B)** Effect of temperature on 5′-FDA yield. The assays were carried out with Tris-HCl (50 mM, pH 7.8), containing 200 mM KF, 1 mM SAM and 50 μg/ml pure enzyme for 0.5 h. Error bars represent the standard deviation obtained from three replicates. ***p < 0.001.

Action temperature is one of the important factors in evaluating the value of enzyme utilization. In order to determine the optimum temperature of the enzymes, Fam and Fme were purified by affinity chromatography and then incubated with SAM and KF at different temperatures (Figure 3A). Maximum activity of Fme was observed at 60°C, which was higher than that of Fam (50°C). The difference in the amount of product produced by the two enzymes at the optimum temperature was then explored (Figure 3B). At 50°C, the purified Fam gave a smaller amount of product than Fme, which was contrary to the comparison between crude enzymes. Further studies showed that imidazole caused irreversible damage to the Fam activity during the purification process, as the elution buffer containing 400 mM imidazole caused greater loss of activity (data not shown). Under optimal conditions, the specific activity of Fam was 0.025 U/mg and that of Fme was 0.082 U/mg.

Up to now, most of the known industrial enzymes come from bacteria and fungi, and usually work under mild reaction conditions (Mathew et al., 2016). It is desirable to enhance the utility of enzymes under extreme conditions by obtaining thermostable enzymes. One method of developing industrial enzymes is to identify novel enzymes from Archaea that usually survive in extreme environments. As the archaea-derived Fme showed a higher optimal temperature, thermal stability studies were then carried out by determining the enzyme residual activity after 30 min of incubation at 30°C or 40°C (Supplementary Figure S4). As expected, Fme showed better thermal stability and maintained about 78% of the relative activity after 30 min of incubation at 40°C, while the relative activity of the most efficient fluorinase (Fxh) maintained 14%. The results showed that Fme could be useful for practical applications.

### 3.4 Site-Directed Mutagenesis

Although more and more enzymes are being used in the industry to produce valuable products, most are limited in terms of stability, catalytic efficiency, or specificity. Site-directed mutagenesis has been proven to be an extremely powerful means to improve the application of enzymes (Yang et al., 2017). In general, the acquisition of a large number of enzyme mutants and rapid screening methods are the keys to the rapid acquisition of effective mutant enzymes.

To speed up the screening efficiency, the mutant catalytic verification was attempted using crude enzyme instead of a purified protein sample. The ability to actually catalyze the conversion of the cell-free extract and the whole-cell extract was compared, and it was found that the whole-cell system showed lower relative activity (Supplementary Figure S5). Markakis et al. (2020) identified that the effusion of fluoride and the permeability of SAM limited the availability of enzymes to substrates in the living cells. Therefore, the assay was carried out using the *E. coli* cell-free extracts.

The strategy of alanine scanning combined with structure analysis was applied to obtain a small library. The structure of FlA has been resolved and shows a hexamer symmetrically formed by two trimers, and the substrate molecule (SAM) is located in the gap between two monomers in the trimer (Dong et al., 2004). Considering that no information about Fam or Fme exists in the current crystal structure database, the protein sequences of these two enzymes were queried on the Swiss-Model platform for computer simulation. Subsequently, the protein template with the highest score was selected for modeling. The results showed that the structure 2V7V of Protein Data Bank was the optimal option for both two proteins and scored higher with Fam (Zhu et al., 2007). Therefore, a homology model of Fam was built for further research.

The strategy of screening hot spot residue based on protein structure has been proven to be effective (Hestericová et al., 2018). To improve the catalytic performance of Fam, 32 amino acids (11F, 12M, 13S, 14D, 15L, 21S, 48W, 72T, 73T, 74T, 75Y, 76P, 77A, 78T, 79G, 84S, 119A, 120P, 122N, 124L, 153T, 154F, 155Y, 156S, 160V, 161A, 209D, 211P, 212F, 214N, 253F, and 268S) within 10 Å with fluorine atoms as the center in the model of Fam were replaced by alanine. Mutants were overexpressed in *E. coli,* and the cells were lysed to obtain cell-free extracts and then incubated with SAM and KF. The evolved fluorinase T72A was first identified as the top mutant with improved 5′-FDA yields. To identify new functional sites associated with catalytic activity, 11 amino acid sites within 10 Å with site 72T as the regional center were replaced by alanine (V41A, G53A, Y56A, I57A, L60A, F70A, Y117A, I118A, L125A, V128A, and S164A). S164A was screened as new effective one from the 11 mutants. Based on the results of the primary selection, saturation mutagenesis was carried out at sites 72T and 164S (Supplementary Figure S6), and three mutants (T72A, T72C, and S164A) were identified that showed higher yields than the wild-type.

**FIGURE 4:**
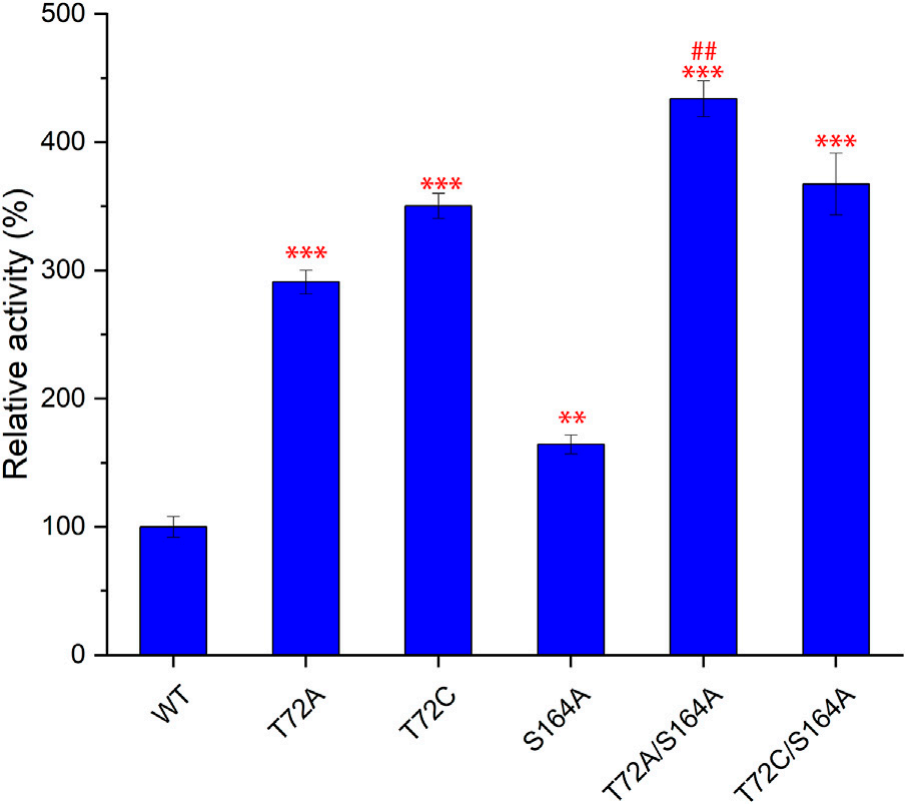
Comparison of catalytic function of different mutants. 5′-FDA was measured after 45 min of incubation at 50°C in the presence of purified enzyme (50 μg/ml). Error bars represent the standard deviation obtained from three replicates. **p < 0.01 vs. WT; ***p < 0.001 vs. WT; # #*p* < 0.01 vs. T72C.

Subsequently, S164A was introduced to T72A and T72C to give T72A/S164A and T72C/S164A. To verify the improvement of the catalytic function, these two combinatorial mutants and three single mutants were purified for *in vitro* validation assays (Figure 4). The mutants T72A showed 1.9-fold increase in 5′-FDA yield compared to the wild-type, while S164A showed a 0.8-fold increase. The T72C showed the highest catalytic activity among single mutants, 3.5 times the wild-type in 5′-FDA yield. As expected, the combinatorial mutation strategy was effective as both double mutants showed higher 5′-FDA yields than the initial single mutation, and the highest conversion was observed in T72A/S164A, which showed a 0.2-fold 5′-FDA yield increase than the best single mutation (T72C).

### 3.5 Simulation Analysis of Mutants

To gain structural insight into the best performing mutations, the changes in amino acid flexibility in the mutant protein system were examined (Supplementary Figure S7). The amino acid flexibility distribution trend of Fam was basically the same before and after the mutation, only the 45–52 and 108–114 regions were quite different, and the one in the T72A/S164A system showed a greater score than that of the WT system. The 45–52 region of Fam was located near the substrate SAM, and the greater flexibility of the mutant was more conducive to the conformational matching of amino acid residues of the substrate and the active center, achieving a stable binding state.

**FIGURE 5:**
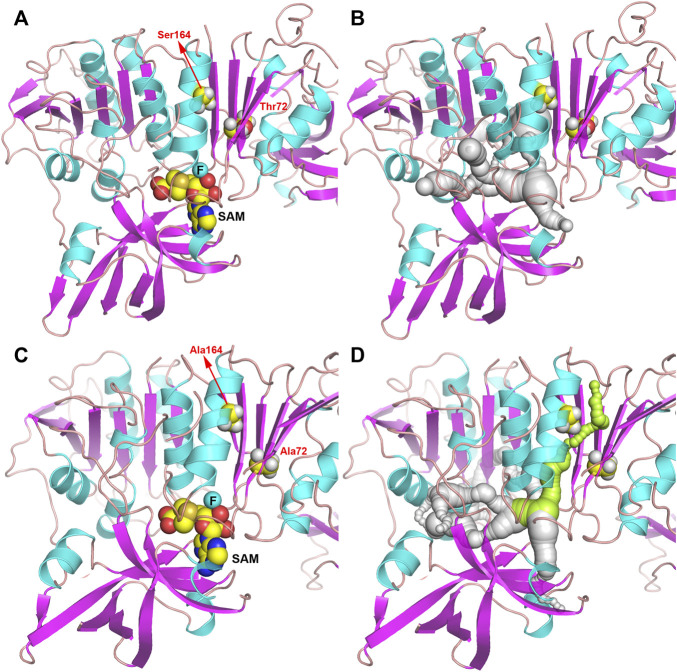
Structure of Fam shown as ribbon. **(A,B)** Fam-WT. **(C,D)** Fam-T72A/S164A. The pathway for the substrate to enter the active site is shown in gray and green.

In order to further study the mechanism of the increase in enzyme activity caused by mutation, the homology models complexed to substrates of wild-type or T72A/S164A were analyzed (Figure 5). There was no obvious change in the overall structure of the protein before and after the mutation. But by comparing the pathways of the F^−^ to enter the active site, it can be found that in the wild-type system, the substrate cannot reach the active center through the gap between T72 and S164. In the T72A/S164A system, the α-helix and β-sheet where Ala164 and Ala72 are located form a channel that can accommodate the passage of the substrate (the green part in Figure 5D). The T72 and S164 are mutated to alanine with a smaller side chain volume and form a channel that can accommodate water molecules and F ions.

### 3.6 Further Mutation Based on Modeling Analysis

Theoretically, replacing alanine with a smaller glycine will further increase the size of the channel. Based on the results of the single mutation, we found that the mutation at amino acid 72 to obtain T72G caused a sharp decrease in activity, whereas the same mutation at amino acid 164 caused only a minor decrease in activity. To further confirm that the formation of a substrate channel increases the enzyme activity, the S164G was adopted to T72A to obtain T72A/S164G. Pure enzyme catalytic experiments proved that mutant T72A/S164G had the highest catalytic activity with a 2.2-fold increase in the catalytic efficiency (*k*_*ca*t_/*K*_*m*_) than the wild-type (Table 1).

**TABLE 1:** Comparative kinetics data of Fam and the variants for the conversion of SAM into 5′-FDA^a^.

Fluorinase	K_m_ [µM]	k_cat_ [min^−1^]	k_cat_/K_m_ [mM^−1^min^−1^]
WT	77.17 ± 14.10	0.171	2.222
T72A/S164G	59.20 ± 17.10	0.428	7.225
T72A/S164A	111.27 ± 9.66	0.432	3.886
T72C/S164G	71.28 ± 9.40	0.253	3.543
T72C/S164A	113.54 ± 11.50	0.303	2.669

a Assays were carried out in Tris-HCl, buffer (50 mM, pH 7.8), containing KF (100 mM), SAM (60–600 µM), and enzyme (50–75 μg/ml) at 50 °C for 10 min.

## 4 Conclusion

In summary, two novel fluorinases were obtained and the corresponding protein sequences were expressed in *E. coli* with the ability to transfer fluoride ions reserved. Fam from *Amycolatopsis* sp. CA-128772 was more similar to the reported fluorinases. Fme was the first archaea-derived fluorinase and showed good thermostability. Considering the credibility of computer modeling, take Fam as an example, site-directed mutagenesis was applied to improve the catalytic effect on natural substrates SAM by the strategy of alanine scan combined with structure analysis. Three single mutants and four double mutants were identified. The best mutant, T72A/S164G, showed a 2.2-fold increase in the catalytic efficiency (*k*_*ca*t_/*K*_*m*_). This research is the first to improve the catalytic activity of fluorinase on natural substrates through engineering means, and it proposes the possibility of fluoride ion channels.

## Data Availability

The original contributions presented in the study are included in the article/Supplementary Material; further inquiries can be directed to the corresponding authors.
