# Supplementary material for: Identification of Two Novel Fluorinases From Amycolatopsis sp. CA-128772 and Methanosaeta sp. PtaU1.Bin055 and a Mutant With Improved Catalytic Efficiency With Native Substrate
Source: Front Bioeng Biotechnol. 2022 Jun 13;10:881326. doi: 10.3389/fbioe.2022.881326 (PMC9234330; doi:10.3389/fbioe.2022.881326)
Supplement: Supplementary file 1 [file DataSheet1.docx]

**Identification of two novel fluorinases from *Amycolatopsis* sp. CA-128772 and *Methanosaeta* sp. PtaU1.Bin055 and a mutant with improved catalytic efficiency with native substrate**

**Xinming Feng^a,b^, Yujin Cao^a^*, Wei Liu^a^*, and Mo Xian^a^***

**^a^ CAS Key Laboratory of Biobased Materials, Qingdao Institute of Bioenergy and Bioprocess Technology, Chinese Academy of Sciences, Qingdao, China**

**^b^ University of Chinese Academy of Sciences, Beijing, China**

**Correspondence:**

**Yujin Cao, Email: caoyj@qibebt.ac.cn**

**Wei Liu, Email: liuwei@qibebet.ac.cn**

**Mo Xian, Email: xianmo1@qibebt.ac.cn**

**HPLC analysis**

The 5’-FDA was detected by HPLC at 260nm using Agilent Poroshell 120 EC-C18 column (2.1*100 mm, 1.9µm) with Shimadzu Ultra High Performance Liquid Chromatography System.

Mobile phase A: acetonitrile; Mobile phase B: water.

Flow rate: 0.2 mL/min.

Elution program: 5% A for 0.6 min; 5-30% A for 4 min; 30% A for 0.4 min; 30-5% A for 1min; 5% A for 4min.

**Statistical analysis**

Origin statistical software was used for data analysis via a two-sample T-test. Differences were considered statistically significant at *p* < 0.05.

**CLUSTAL O (1.2.4) multiple sequence alignment**

OPY51785.1 MLQNTKNEYKPRDGGGHRPIIGFMSDLGTEDDSVGICKGVMLGLCPDAVIVDISHSMTPW60

SDK90789.1 -----------MSDSYSRPIIAFMSDLGTTDDSVAQCKGLMMSICQDVTVVDVCHSMEPW 49

WP_192883607.1 ----------MSADPTQRPIIGFMSDLGTTDDSVAQCKGLMHSICPGVTVIDVCHSMTPW 50

Q70GK9.1 ----------MAANSTRRPIIAFMSDLGTTDDSVAQCKGLMYSICPDVTVVDVCHSMTPW 50

pdb|5LMZ|A -------GAMVAANGSQRPIIAFMSDLGTTDDSVAQCKGLMHSICPGVTVVDVCHSMTPW 53

WP_015619887.1 -----------------------MSDLGTTDDSVAQCKGLMLSICPGVTIVDVNHSMTPW 37

WP_103354124.1 ------------MAKPSRPIIAFMSDLGITDDSVAQCKGLMLSVCPDVTIVDVCHTMKPW 48

WP_014985135.1 -----------------------MSDLGITDDSVAQCKGLMLSVCPDVTIVDICHTMQPW 37

***** ****. ***:* .:* ...::*: *:* **

OPY51785.1 DIDQGSRLIVDLPKFFPNWTTFATTSYRETGTSARSVAIKLPS----------------- 103

SDK90789.1 NVEEGARYIVDLPRFFPEGTVFATTTYPATGTTARSVAVRIKYPAKGGARGQWAGSGEGF 109

WP_192883607.1 DVEEGARYIVDLPRFFPEGTVFATTTYPATGTETRSVAVRIKQAAKGGARGQWAGSAGGF 110

Q70GK9.1 DVEEGARYIVDLPRFFPEGTVFATTTYPATGTTTRSVAVRIKQAAKGGARGQWAGSGAGF 110

pdb|5LMZ|A DVEEGARYIVDLPRFFPEGTVFATTTYPATGTTTRSVAVRIRQAAKGGARGQWAGSGDGF 113

WP_015619887.1 DVEEGARYIVDLPRFFPEGTVFATTTYPATGTATRSVALRIKQAAQGGARGQWAGSGAGF 97

WP_103354124.1 DVEEGARYIVDLPRLFPEGTVFATTTYPATGTTTRSVALRIKQAAKGGARGQWAGSGAGF 108

WP_014985135.1 DVEEGARYIVDLPRLFPEGTVFATTTYPATGTTARSVALRIAHASKGGARGQWAGSGAGF 97

::::*:* *****::**: *.****:* *** :****:::

OPY51785.1 -----GHVYVAPNNGLLTRVIEDHGYVEAYEVTTVGAIPAEPEPTWFSRDMVAYPAAAIA 158

SDK90789.1 ERSEGSYIYIAPNNGLLTTVLQEHGYTEAYEVSSTDVVPARPEPTFYSREMVAIPSAHLA 169

WP_192883607.1 ERAEGSYIYVAPNNGLLTTVLEEHGYIEAYEVSSTKVIPERPEPTFYSREMVAIPAAHLA 170

Q70GK9.1 ERAEGSYIYIAPNNGLLTTVLEEHGYLEAYEVTSPKVIPEQPEPTFYSREMVAIPSAHLA 170

pdb|5LMZ|A ERADGSYIYIAPNNGLLTTVLEEHGYIEAYEVTSTKVIPANPEPTFYSREMVAIPSAHLA 173

WP_015619887.1 ERAEGSYIYIAPNNGLLTTVIEEHGYIEAYEVSNTKVIPAEPEPTFYSREMVAIPSAHLA 157

WP_103354124.1 ERAEGSYIYIAPNNGLLTSVIEEHGYVEAYEVSSTEVIPEQPEPTFYSREMVALPSAHLA 168

WP_014985135.1 ERKEGSYIYIAPNNGLLTTVIKEHGYLEAYEVSSPEVIPEQPEPTFYSREMVALPSAHLA 157

.::*:******** *:::*** *****:. .:* .****::**:*** *:* :*

OPY51785.1 AGFPLENVGRPLNDSEIVRADLPRYTQAEDGIIQGIVTTIDRPFGNIWTNIPRRIIENEL 218

SDK90789.1 AGYPLEKVGRKLQDSEIVRFTPPQATVSPEGDLSGVVTAIDHPFGNIWTSIHRDNLE-SA 228

WP_192883607.1 AGFPLSEVGRPLEDSEIVRYQPPQVE-ISGDTLTGVVSAIDHPFGNVWTNIHRTHLE-KA 228

Q70GK9.1 AGFPLSEVGRPLEDHEIVRFNRPAVE-QDGEALVGVVSAIDHPFGNVWTNIHRTDLE-KA 228

pdb|5LMZ|A AGFPLAEVGRRLDDSEIVRFHRPAVE-ISGEALSGVVTAIDHPFGNIWTNIHRTDLE-KA 231

WP_015619887.1 AGFPLNEVGRALSDDEIVRFAKPKPSTVSGGVLSGVITNIDHPFGNLWTNIHRTDLE-KA 216

WP_103354124.1 AGFPLEKVGRPLADDEIVRFERAKPAQNDDGELVGVVTAIDHPFGNVWTNIHREDLE-KL 227

WP_014985135.1 AGFPLEKVGRRLADDEIVRFERKDPELVADHDLVGYVTNIDHPFGNVWTNIHRTDLE-KL 216

**:** :*** * * **** : * :: **:****:**.* * :* .

OPY51785.1 RIAYGTNIRVVLDNLLVLEVPFMRTFGDVGL-KKPMCYINSRGYFSLAYYGGNLADPYNI 277

SDK90789.1 GVGYGTNLKIVLDDVFPFELPLSPTFADAGEVGDPVVYVNSRGYLSLARNAASLAYPYNL 288

WP_192883607.1 GIGYGKRIKIILDDVLPFEQTLVPTFADAGEIGGVAAYLNSRGYLSLARNAASLAYPFNL 288

Q70GK9.1 GIGYGARLRLTLDGVLPFEAPLTPTFADAGEIGNIAIYLNSRGYLSIARNAASLAYPYHL 288

pdb|5LMZ|A GIGQGKHLKIILDDVLPFEAPLTPTFADAGAIGNIAFYLNSRGYLSLARNAASLAYPYNL 291

WP_015619887.1 GIGYQTQLRLLLDGVLTFDLPLVPTFADAGQIGDPVIYINSRGYLALARNAAPLAYPYNL 276

WP_103354124.1 GAGYGTRLRITLDEVLPFDLPLSPTFADAGPIGTPVAYLSSRGYLALARNAASLAYPYNL 287

WP_014985135.1 GVGYGTKLRITLDGVLPFELPLSPTFADAGEIGAAVAYLSSRGYLALARNAASLAYPYNL 276

. .::: ** :: :: : **.*.* *:.****:::* .. ** *:::

OPY51785.1 RRGMPVKIESITR 290

SDK90789.1 KEGMSVRVTRS-- 299

WP_192883607.1 KAGLKVRVETN-- 299

Q70GK9.1 KEGMSARVEAR-- 299

pdb|5LMZ|A KAGLKVRVEAR-- 302

WP_015619887.1 KAGLTVTVTKA-- 287

WP_103354124.1 NAGISVRVVAA-- 298

WP_014985135.1 KAGISVQVKVG-- 287

. *: . :

The amino acids labeled red were screened in the first round within 10 angstroms of 5’-FDA in the Fam model established with 2V7V as the template. pdb|5LMZ|A: Fluorinase from *Streptomyces sp*. MA37. WP_192883607.1: Fluorinase from *Streptomyces xinghaiensis*. Q70GK9.1: Fluorinase from *Streptomyces cattleya*. WP_015619887.1: Fluorinase from *Actinoplanes* sp. N902-109. WP_014985135.1: Fluorinase from *Nocardia brasiliensis.* SDK90789.1: Fluorinase from *Actinopolyspora mzabensis*. WP_103354124.1: Fam. OPY51785.1: Fme.

**Amino acid screening of the second round of** **mutation**

MAKPSRPIIAFMSDLGITDDSVAQCKGLMLSVCPDVTIVDVCHTMKPWDVEEGARYIVDLPRLFPEGTVF

ATTTYPATGTTTRSVALRIKQAAKGGARGQWAGSGAGFERAEGSYIYIAPNNGLLTSVIEEHGYVEAYEV

SSTEVIPEQPEPTFYSREMVALPSAHLAAGFPLEKVGRPLADDEIVRFERAKPAQNDDGELVGVVTAIDH

PFGNVWTNIHREDLEKLGAGYGTRLRITLDEVLPFDLPLSPTFADAGPIGTPVAYLSSRGYLALARNAAS

LAYPYNLNAGISVRVVAA

The amino acids labeled yellow were within the location of 7 angstroms in the Fam model established with 2V7V as the template

**Table S1 List of primers.**

| Name | Primer (5’→3’) |
| --- | --- |
| F11A-F | TTGCGgctTTTATGAGCGATCTGGGCATTACC |
| F11A-R | GCTCATAAAagcCGCAATAATCGGGCGGCTAG |
| M12A-F | TGCGTTTgctAGCGATCTGGGCATTACCGATG |
| M12A-R | GATCGCTagcAAACGCAATAATCGGGCGGCTA |
| S13A-F | GTTTATGgctGATCTGGGCATTACCGATGATAG |
| S13A-R | CCAGATCagcCATAAACGCAATAATCGGGCGG |
| D14A-F | TTTATGAGCgctCTGGGCATTACCGATGATAGCG |
| D14A-R | CCCAGagcGCTCATAAACGCAATAATCGGGCG |
| L15A-F | GAGCGATgctGGCATTACCGATGATAGCGTGG |
| L15A-R | TAATGCCagcATCGCTCATAAACGCAATAATCG |
| S21A-F | ATGATgctGTGGCGCAATGCAAAGGCCTGATG |
| S21A-R | TTGCGCCACagcATCATCGGTAATGCCCAGATCG |
| W48A-F | TGAAACCGgctGATGTGGAAGAAGGCGCGCGC |
| W48A-R | CACATCagcCGGTTTCATGGTATGGCACACAT |
| T72A-F | TTTGCGgctACCACCTATCCTGCGACCGGTAC |
| T72A-R | TAGGTGGTagcCGCAAACACGGTGCCTTCAGG |
| T73A-F | TTGCGACCgctACCTATCCTGCGACCGGTACC |
| T73A-R | ATAGGTagcGGTCGCAAACACGGTGCCTTCAG |
| T74A-F | TTGCGACCACCgctTATCCTGCGACCGGTACCAC |
| T74A-R | ATAagcGGTGGTCGCAAACACGGTGCCTTCAG |
| Y75A-F | TTGCGACCACCACCgctCCTGCGACCGGTACCACTAC |
| Y75A-R | agcGGTGGTGGTCGCAAACACGGTGCCTTCAG |
| P76A-F | ACCACCTATgctGCGACCGGTACCACTACTCG |
| P76A-R | GTCGCagcATAGGTGGTGGTCGCAAACACGGT |
| T78A-F | TATCCTGCGgctGGTACCACTACTCGTTCAGTTGCG |
| T78A-R | GTACCagcCGCAGGATAGGTGGTGGTCGCAAA |
| G79A-F | CgctACCACTACTCGTTCAGTTGCGCTGCGCA |
| G79A-R | AACGAGTAGTGGTagcGGTCGCAGGATAGGTGGTGG |
| S84A-F | ACTACTCGTgctGTTGCGCTGCGCATTAAACA |
| S84A-R | GCAACagcACGAGTAGTGGTACCGGTCGCAGG |
| P120A-F | TATTGCGgctAACAACGGCTTACTGACCAGCG |
| P120A-R | CGTTGTTagcCGCAATATAAATATAGCTGCCTTCC |
| N122A-F | GAACgctGGCTTACTGACCAGCGTGATTGAAG |
| N122A-R | TCAGTAAGCCagcGTTCGGCGCAATATAAATATAGCTG |
| L124A-F | AACAACGGCgctCTGACCAGCGTGATTGAAGAACA |
| L124A-R | GTCAGagcGCCGTTGTTCGGCGCAATATAAAT |
| T153A-F | CGGAACCGgctTTTTATAGCCGCGAAATGGTG |
| T153A-R | ATAAAAagcCGGTTCCGGCTGTTCCGGAATCA |
| F154A-F | AACCGACCgctTATAGCCGCGAAATGGTGGCA |
| F154A-R | GCTATAagcGGTCGGTTCCGGCTGTTCCGGAA |
| Y155A-F | CTTTgctAGCCGCGAAATGGTGGCATTACCGA |
| Y155A-R | TTTCGCGGCTagcAAAGGTCGGTTCCGGCTGT |
| S156A-F | CCTTTTATgctCGCGAAATGGTGGCATTACCG |
| S156A-R | TTCGCGagcATAAAAGGTCGGTTCCGGCTGTT |
| V160A-F | CGAAATGgctGCATTACCGAGCGCGCATTTAG |
| V160A-R | GTAATGCagcCATTTCGCGGCTATAAAAGGTC |
| D209A-F | GCGATTgctCATCCGTTTGGCAACGTGTGGAC |
| D209A-R | AACGGATGagcAATCGCGGTCACAACACCCAC |
| P211A-F | TCATgctTTTGGCAACGTGTGGACCAACATTC |
| P211A-R | CGTTGCCAAAagcATGATCAATCGCGGTCACAAC |
| F212A-F | ATTGATCATCCGgctGGCAACGTGTGGACCAACA |
| F212A-R | CCagcCGGATGATCAATCGCGGTCACAACACC |
| N214A-F | ATCATCCGTTTGGCgctGTGTGGACCAACATTCATCGC |
| N214A-R | agcGCCAAACGGATGATCAATCGCGGTCACAA |
| F253A-F | CCCTACTgctGCAGATGCGGGCCCTATTGGTA |
| F253A-R | CATCTGCagcAGTAGGGCTCAGAGGCAGATCA |
| S268A-F | TATTTAAGCgctCGCGGCTATTTAGCGCTGGC |
| S268A-R | CCGCGagcGCTTAAATACGCCACAGGAGTACC |
| V41A-F | TGTGGATgctTGCCATACCATGAAACCGTGGG |
| V41A-R | TATGGCAagcATCCACAATGGTCACATCCGGG |
| G53A-F | AAGAAgctGCGCGCTATATTGTTGATCTGCCG |
| G53A-R | ATAGCGCGCagcTTCTTCCACATCCCACGGTTT |
| Y56A-F | CGCGCgctATTGTTGATCTGCCGCGCTTATTT |
| Y56A-R | ATCAACAATagcGCGCGCGCCTTCTTCCACAT |
| I57A-F | GCGCTATgctGTTGATCTGCCGCGCTTATTTC |
| I57A-R | GATCAACagcATAGCGCGCGCCTTCTTCCACA |
| L60A-F | ATTGTTGATgctCCGCGCTTATTTCCTGAAGG |
| L60A-R | CGCGGagcATCAACAATATAGCGCGCGCCTTC |
| F70A-F | TGAAGGCACCGTGgctGCGACCACCACCTATCCTGC |
| F70A-R | CagcCACGGTGCCTTCAGGAAATAAGCGCGGC |
| Y117A-F | ATATTgctATTGCGCCGAACAACGGCTTACTG |
| Y117A-R | CGGCGCAATagcAATATAGCTGCCTTCCGCACG |
| I118A-F | TATTTATgctGCGCCGAACAACGGCTTACTGA |
| I118A-R | TCGGCGCagcATAAATATAGCTGCCTTCCGCAC |
| L125A-F | AACGGCTTAgctACCAGCGTGATTGAAGAACATG |
| L125A-R | CTGGTagcTAAGCCGTTGTTCGGCGCAATATA |
| V128A-F | GACCAGCgctATTGAAGAACATGGCTATGTGGAA |
| V128A-R | CTTCAATagcGCTGGTCAGTAAGCCGTTGTTC |
| S164A-F | ATTACCGgctGCGCATTTAGCAGCGGGCTTTC |
| S164A-R | AATGCGCagcCGGTAATGCCACCATTTCGCGG |
| T72F-F | GTTTGCGtttACCACCTATCCTGCGACCGGTA |
| T72F-R | AGGTGGTaaaCGCAAACACGGTGCCTTCAGGA |
| T72L-F | GTTTGCGttaACCACCTATCCTGCGACCGGTA |
| T72L-R | AGGTGGTtaaCGCAAACACGGTGCCTTCAGGA |
| T72I-F | GTTTGCGattACCACCTATCCTGCGACCGGTA |
| T72I-R | AGGTGGTaatCGCAAACACGGTGCCTTCAGGA |
| T72M-F | GTTTGCGatgACCACCTATCCTGCGACCGGTA |
| T72M-R | AGGTGGTcatCGCAAACACGGTGCCTTCAGGA |
| T72V-F | GTTTGCGgttACCACCTATCCTGCGACCGGTA |
| T72V-R | AGGTGGTaacCGCAAACACGGTGCCTTCAGGA |
| T72S-F | GTTTGCGtctACCACCTATCCTGCGACCGGTA |
| T72S-R | AGGTGGTagaCGCAAACACGGTGCCTTCAGGA |
| T72P-F | TTTGCGcctACCACCTATCCTGCGACCGGTAC |
| T72P-R | TAGGTGGTaggCGCAAACACGGTGCCTTCAGG |
| T72Y-F | GTTTGCGtatACCACCTATCCTGCGACCGGTA |
| T72Y-R | AGGTGGTataCGCAAACACGGTGCCTTCAGGA |
| T72H-F | GTTTGCGcatACCACCTATCCTGCGACCGGTA |
| T72H-R | AGGTGGTatgCGCAAACACGGTGCCTTCAGGA |
| T72Q-F | GTTTGCGcaaACCACCTATCCTGCGACCGGTA |
| T72Q-R | AGGTGGTttgCGCAAACACGGTGCCTTCAGGA |
| T72N-F | GTTTGCGaatACCACCTATCCTGCGACCGGTA |
| T72N-R | AGGTGGTattCGCAAACACGGTGCCTTCAGGA |
| T72K-F | GTTTGCGaaaACCACCTATCCTGCGACCGGTA |
| T72K-R | AGGTGGTtttCGCAAACACGGTGCCTTCAGGA |
| T72D-F | GTTTGCGgatACCACCTATCCTGCGACCGGTA |
| T72D-R | AGGTGGTatcCGCAAACACGGTGCCTTCAGGA |
| T72E-F | GTTTGCGgaaACCACCTATCCTGCGACCGGTA |
| T72E-R | AGGTGGTttcCGCAAACACGGTGCCTTCAGGA |
| T72C-F | GTTTGCGtgtACCACCTATCCTGCGACCGGTA |
| T72C-R | AGGTGGTacaCGCAAACACGGTGCCTTCAGGA |
| T72W-F | TTTGCGtggACCACCTATCCTGCGACCGGTAC |
| T72W-R | TAGGTGGTccaCGCAAACACGGTGCCTTCAGG |
| T72R-F | TTTGCGcgtACCACCTATCCTGCGACCGGTAC |
| T72R-R | TAGGTGGTacgCGCAAACACGGTGCCTTCAGG |
| T72G-F | TTTGCGggtACCACCTATCCTGCGACCGGTAC |
| T72G-R | TAGGTGGTaccCGCAAACACGGTGCCTTCAGG |
| S164F-F | CATTACCGtttGCGCATTTAGCAGCGGGCTTT |
| s164F-R | ATGCGCaaaCGGTAATGCCACCATTTCGCGGC |
| S164Y-F | CATTACCGtatGCGCATTTAGCAGCGGGCTTT |
| S164Y-R | ATGCGCataCGGTAATGCCACCATTTCGCGGC |
| S164C-F | ATTACCGtgtGCGCATTTAGCAGCGGGCTTTC |
| S164C-R | AATGCGCacaCGGTAATGCCACCATTTCGCGG |
| S164L-F | CATTACCGttaGCGCATTTAGCAGCGGGCTTT |
| S164L-R | ATGCGCtaaCGGTAATGCCACCATTTCGCGGC |
| S164P-F | ATTACCGcctGCGCATTTAGCAGCGGGCTTTC |
| S164P-R | AATGCGCaggCGGTAATGCCACCATTTCGCGG |
| S164H-F | ATTACCGcatGCGCATTTAGCAGCGGGCTTTC |
| S164H-R | AATGCGCatgCGGTAATGCCACCATTTCGCGG |
| S164Q-F | ATTACCGcaaGCGCATTTAGCAGCGGGCTTTC |
| S164Q-R | AATGCGCttgCGGTAATGCCACCATTTCGCGG |
| S164W-F | ATTACCGtggGCGCATTTAGCAGCGGGCTTTC |
| S164W-R | AATGCGCccaCGGTAATGCCACCATTTCGCGG |
| S164R-F | ATTACCGcgtGCGCATTTAGCAGCGGGCTTTC |
| S164R-R | AATGCGCacgCGGTAATGCCACCATTTCGCGG |
| S164I-F | CATTACCGattGCGCATTTAGCAGCGGGCTTT |
| S164I-R | ATGCGCaatCGGTAATGCCACCATTTCGCGGC |
| S164M-F | ATTACCGatgGCGCATTTAGCAGCGGGCTTTC |
| S164M-R | AATGCGCcatCGGTAATGCCACCATTTCGCGG |
| S164T-F | ATTACCGactGCGCATTTAGCAGCGGGCTTTC |
| S164T-R | AATGCGCagtCGGTAATGCCACCATTTCGCGG |
| S164N-F | CATTACCGaatGCGCATTTAGCAGCGGGCTTT |
| S164N-R | ATGCGCattCGGTAATGCCACCATTTCGCGGC |
| S164K-F | CATTACCGaaaGCGCATTTAGCAGCGGGCTTT |
| S164K-R | ATGCGCtttCGGTAATGCCACCATTTCGCGGC |
| S164D-F | ATTACCGgatGCGCATTTAGCAGCGGGCTTTC |
| S164D-R | AATGCGCatcCGGTAATGCCACCATTTCGCGG |
| S164E-F | ATTACCGgaaGCGCATTTAGCAGCGGGCTTTC |
| S164E-R | AATGCGCttcCGGTAATGCCACCATTTCGCGG |
| S164G-F | ATTACCGggtGCGCATTTAGCAGCGGGCTTTC |
| S164G-R | AATGCGCaccCGGTAATGCCACCATTTCGCGG |
| S164V-F | ATTACCGgttGCGCATTTAGCAGCGGGCTTTC |
| S164V-R | AATGCGCaacCGGTAATGCCACCATTTCGCGG |

**Table S2** **Deduced functions of ORFs in the putative fluorometabolite biosynthetic gene cluster in *Amycolatopsis* sp. CA-128772** **compared with the homologs in *S. cattleya***

| **ORF** | **Proposed functions** | **AA** | **Identity** | **Homologue in S. cattleya** |
| --- | --- | --- | --- | --- |
| FlB5 | 5'-FDA phosphorylase | 292 | 56% | FlB |
| FlI5 | S-adenosyl-L-homocysteine hydrolase | 480 | 75% | FlI |
| Fam | 5'-FDA synthase | 298 | 83% | FlA |
| FlH5 | Sodium/hydrogen exchanger family | 464 | 47% | FlH |
| FlG5 | Bacterial regulatory proteins | 225 | 45% | FlG |
| FlL5 | Bacterial regulatory proteins | 220 | 31% | FlL |
| FlJ5 | Bacterial regulatory proteins | 342 | 36% | FlJ |
| FlF5 | Bacterial regulatory proteins | 191 | 59% | FlF |

**Table S3** **Deduced functions of ORFs in the putative fluorometabolite biosynthetic gene cluster in *Methanosaeta* sp. PtaU1.Bin055** **compared with the homologs in *S. cattleya***

| **ORF** | **Proposed functions** | **AA** | **Identity** | **Homologue in** **S. cattleya** |
| --- | --- | --- | --- | --- |
| Fme | 5'-FDA synthase | 250 | 50% | FlA |
| FlK6 | Fluoroacetyl-CoA thioesterase | 114 | 50% | FlK |

**Supplementary Figures**


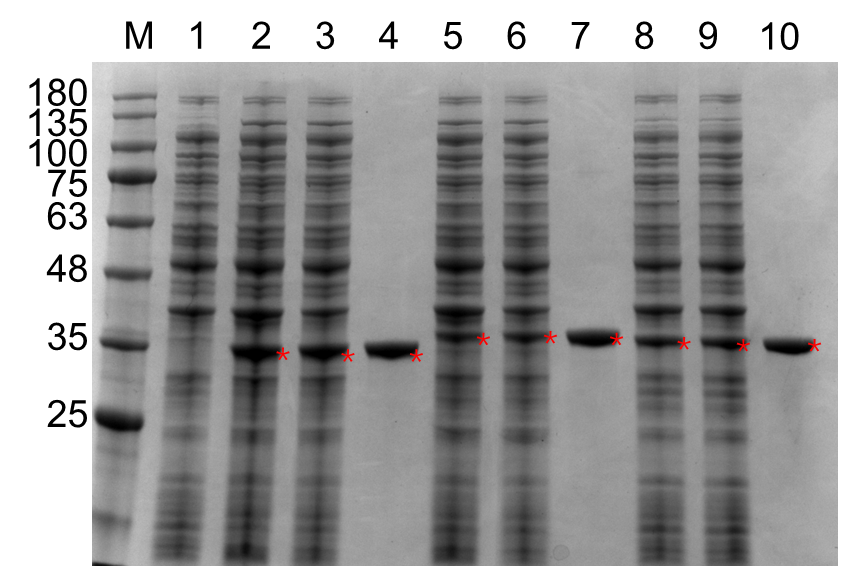


**Figure S1.** SDS-PAGE for BL21(DE3) over expression of the Fme, Fam or FlA. M: marker; 1: control; 2: whole cells expressing Fme; 3: soluble fraction of Fme; 4: pure Fme; 5: whole cells expressing Fam; 6: soluble fraction of Fam; 7: pure Fam; 8: whole cells expressing FlA; 9: soluble fraction of FlA; 10: pure FlA


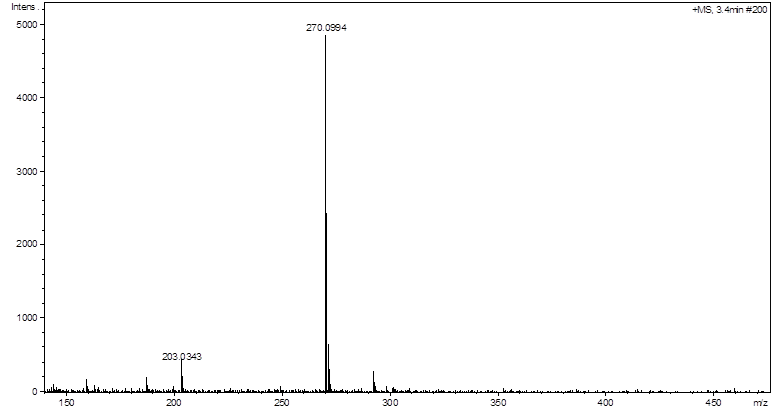


**Figure S2.** The LC-HRMS analysis of 5’-FDA (retention time: 3.4 min; m/z=270.0). Liquid chromatography high-resolution mass spectrometry (LC-HRMS) analysis was performed on an Agilent Technologies 6520 Accurate-Mass Q-TOF LC–MS instrument with an Agilent Eclipse Plus C18 column (4.6×100 mm). LC detection procedure: 15% CH_3_CN in H_2_O from 0 to 5 min, 15% to 95% CH_3_CN in H_2_O from 5 to 12 min, 95% CH_3_CN in H_2_O from 12 to 15 min, 95% to 15% CH_3_CN in H2O from 15 to 18 min, 15% CH_3_CN in H_2_O from 18 to 20 min (H_2_O and CH_3_CN containing 0.1% formic acid (vol/vol)). The flow rate of 0.2 mL/min was used.


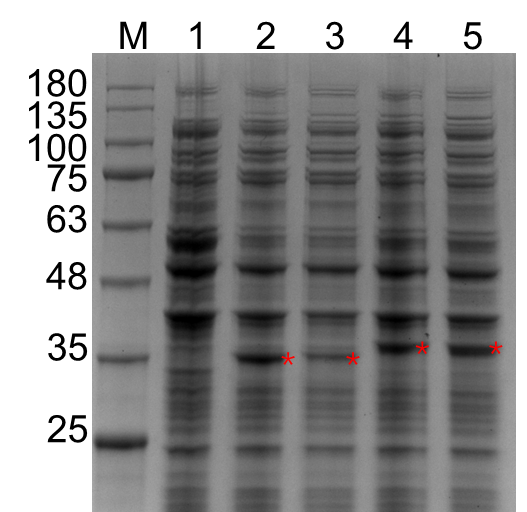


**Figure S3**. SDS-PAGE for BL21(DE3) over expression of the Fam0 and Fam. M: marker; 1: control; 2: whole cells expressing Fam0; 3: soluble fraction of Fam0; 4: whole cells expressing Fam; 5: soluble fraction of Fam


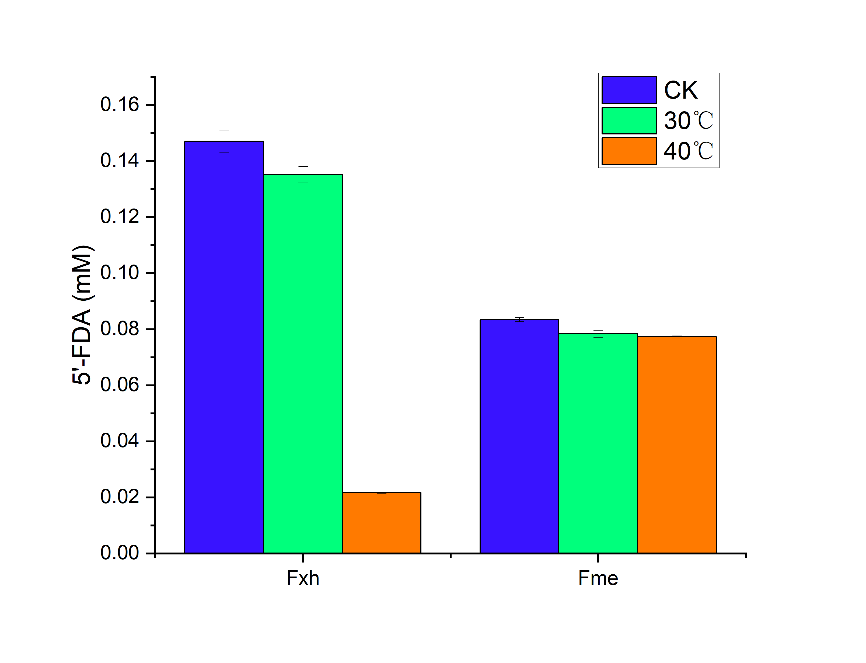


**Figure S4.** The influence of temperature on fluorinase stability. Purified enzymes were incubated at different temperatures for 30 min before the assays were carried out with Tris-HCl (50 mM, pH7.8) containing 200 mM KF, 1 mM SAM and 50 µg/mL pure enzyme at 50 °C for 45 min. Error bars represent the standard deviation obtained from 3 replicates.

**Figure S5.** Comparison of catalytic function of whole cell and crude enzyme solution.

5’-FDA was measured after 1.5 h incubation at 50 °C in the presence of whole-cell or cell-free extract.


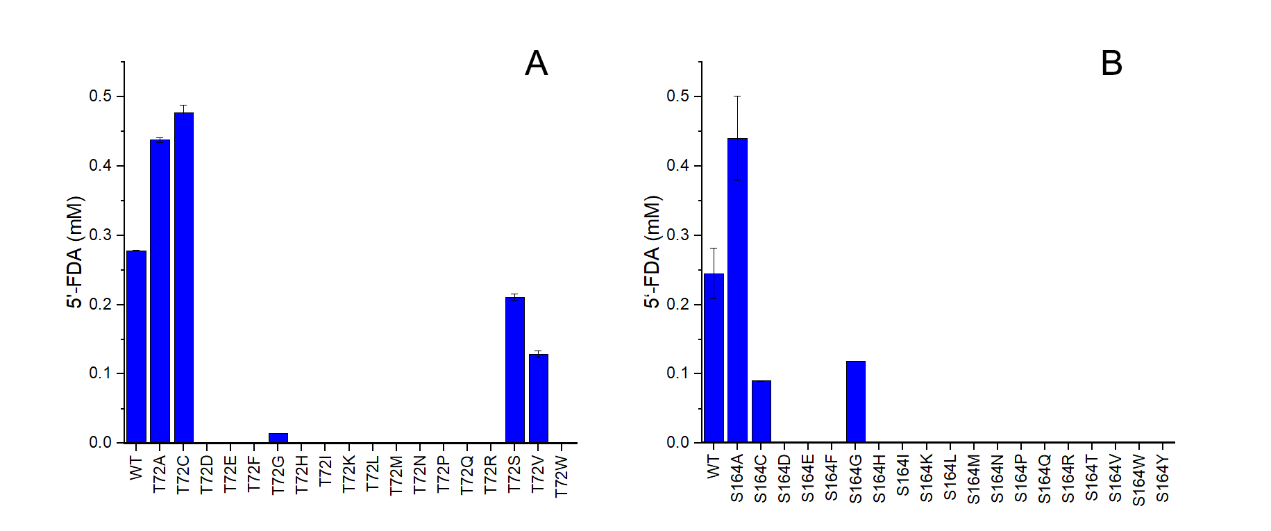


**Figure S6.** Comparison of catalytic function of saturated Fam mutants at 72 amino acid point (A) and 164 amino acid point. 5’-FDA was measured after 1.5 h incubation at 50 °C in the presence of cell free extracts. Error bars represent the standard deviation obtained from 2 replicates.


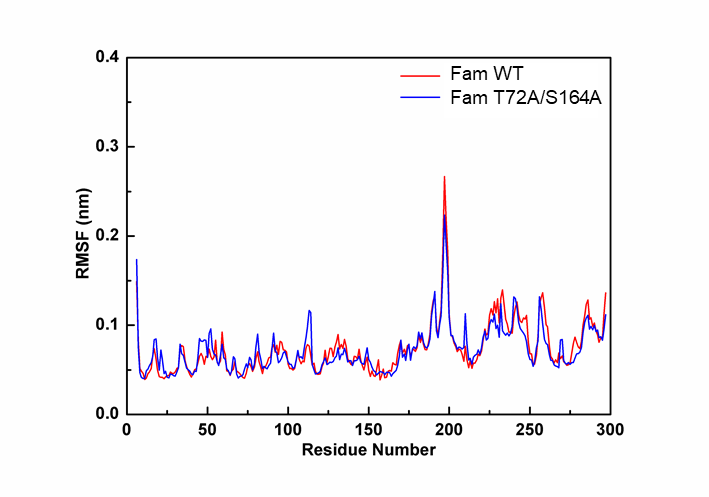


**Figure S7.** Root mean square fluctuation (RMSF) of the Fam and the T72A/S164A mutant.


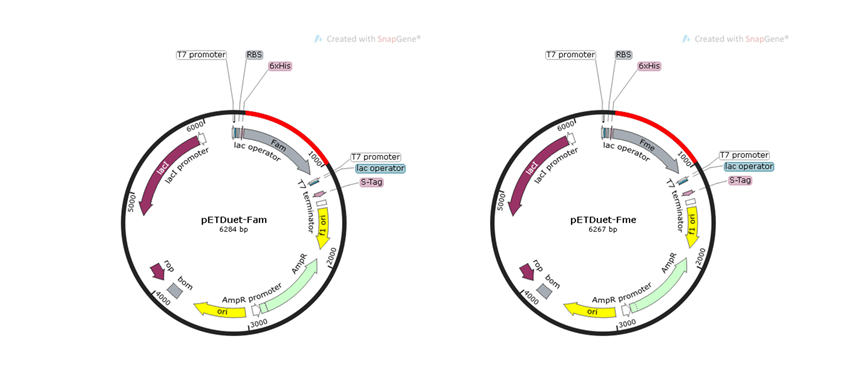


**Figure S8.** The plasmid map of pETDuet-Fam and pETDuet-Fme


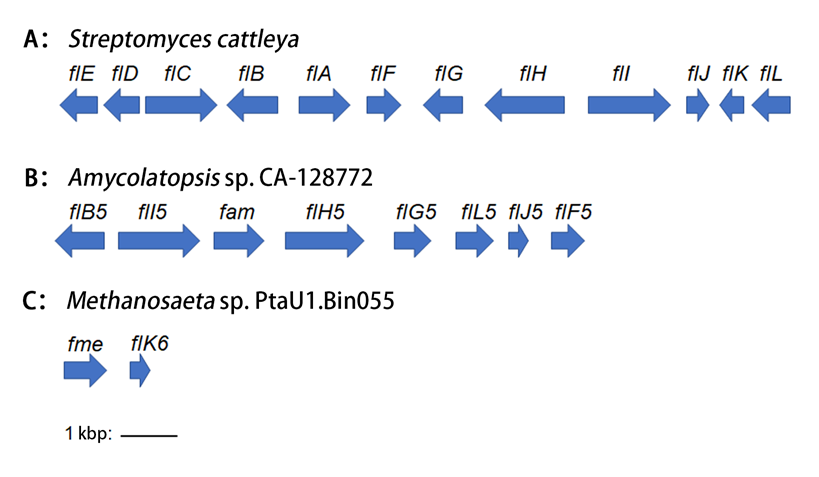


**Figure S9.** Organisation of genes around the fluorinase generated by FramePlot 4.0beta (nocardia.nih.go.jp)
